# Individualized versus Standardized Risk Assessment in Patients at High Risk for Adverse Drug Reactions (The IDrug Randomized Controlled Trial)–Never Change a Running System?

**DOI:** 10.3390/ph14101056

**Published:** 2021-10-18

**Authors:** Katja S. Just, Catharina Scholl, Miriam Boehme, Kathrin Kastenmüller, Johannes M. Just, Markus Bleckwenn, Stefan Holdenrieder, Florian Meier, Klaus Weckbecker, Julia C. Stingl

**Affiliations:** 1 Institute of Clinical Pharmacology, University Hospital RWTH Aachen, 52074 Aachen, Germany; kjust@ukaachen.de; 2 Research Department, Federal Institute for Drugs and Medical Devices, 53175 Bonn, Germany; catharina.scholl@bfarm-research.de (C.S.); miriam.boehme@bfarm-research.de (M.B.); 3 Max-Planck Research Group, Würzburg Institute of Systems Immunology, University of Würzburg, 97078 Würzburg, Germany; kathrin.kastenmueller@uni-wuerzburg.de; 4 Department of General Practice and Interprofessional Care, Medical Faculty of the University of Witten/Herdecke, 58448 Witten, Germany; johannes.just@uni-wh.de (J.M.J.); klaus.weckbecker@uni-wh.de (K.W.); 5 Department of General Practice, Medical Faculty, Leipzig University, 04103 Leipzig, Germany; markus.bleckwenn@medizin.uni-leipzig.de; 6 Institute of Clinical Chemistry and Clinical Pharmacology, University Hospital Bonn, 53127 Bonn, Germany; stefan.holdenrieder@uni-bonn.de; 7 Institute of Laboratory Medicine, German Heart Centre Munich, Technical University Munich, 80636 Munich, Germany; 8 Wilhelm Loehe University of Applied Sciences (WLH), 90763 Fürth, Germany; florian.meier@wlh-fuerth.de

**Keywords:** adverse drug reactions, pharmacogenetics, pharmacogenomics, personalized medicine, phenprocoumon, DOACs, older adults, bleeding, thromboembolism

## Abstract

The aim of this study was to compare effects of an individualized with a standardized risk assessment for adverse drug reactions to improve drug treatment with antithrombotic drugs in older adults. A randomized controlled trial was conducted in general practitioner (GP) offices. Patients aged 60 years and older, multi-morbid, taking antithrombotic drugs and at least one additional drug continuously were randomized to individualized and standardized risk assessment groups. Patients were followed up for nine months. A composite endpoint defined as at least one bleeding, thromboembolic event or death reported via a trigger list was used. Odds ratios (OR) and 95% confidence intervals (CI) were calculated. In total, *N* = 340 patients were enrolled from 43 GP offices. Patients in the individualized risk assessment group met the composite endpoint more often than in the standardized group (OR 1.63 [95%CI 1.02–2.63]) with multiple adjustments. The OR was higher in patients on phenprocoumon treatment (OR 1.99 [95%CI 1.05–3.76]), and not significant on DOAC treatment (OR 1.52 [95%CI 0.63–3.69]). Pharmacogenenetic variants of CYP2C9, 2C19 and VKORC1 were not observed to be associated with the composite endpoint. The results of this study may indicate that the time point for implementing individualized risk assessments is of importance.

## 1. Introduction

Personalized medicine is meant to improve efficacy and safety of drug treatment. However, strategies to modify drug treatment based on individual treatment risks are sparse. Older adults are often affected by adverse drug reactions (ADR) potentially leading to health emergencies [1,2]. It is estimated that around 6.5% of all admissions to the emergency department are caused by ADRs, mostly concerning older, multi-medicated adults [3]. Focusing only on older adults, even 8.7% of hospital admissions could be attributed to ADRs [4]. The prevalence might be even higher with rising age and drug intake [5]. While the role of potentially inappropriate medication for those admissions is not fully clear, ADRs are in general considered to be preventable [4]. Thereby, older adults often present bleeding ADR events due to antithrombotic drug treatment, with increasing risk at higher age [6]. Balancing the individual bleeding risk might be a fragile, challenging process in older adults. Therefore, older adults would probably benefit mostly from respecting the individual risk for ADRs.

While different antithrombotic drug treatments are available, the benefit–risk ratio in general needs to be balanced between preventing thromboembolic events without substantially increasing the risk for major bleedings. In the case of vitamin-K-antagonists (VKA), precise treatment goals are defined using international normalized ratios (INR), as the risk of bleeding increases not only with age, but also with the achieved intensity of coagulation [7]. However, also directly acting oral anticoagulants (DOAC), acetylsalicylic acid (ASA) and P2Y12-inhibitors such as clopidogrel, alone or in combination expose patients to a risk of bleeding [8,9].

Pharmacogenetics (PGx) is considered to smoothen the way to personalized medicine by improving drug efficacy as well as drug safety [10]. While around 80% of ADRs are considered to be dose-related, the individual drug metabolism affecting effective dose exposures might be in particular of importance for drug safety [11]. Heritable genetic variants can individually modify VKA drug effects through its target vitamin K epoxide reductase (VKORC1) and the metabolizing enzyme cytochrome P450 (CYP) 2C9. While PGx variability clearly impacts on drug effects of the VKA warfarin and dosing-guidelines exist [12], phenprocoumon is the VKA most commonly prescribed in Germany and other European countries [13]. However, using INR measurements the phenprocoumon treatment often gets empirically adjusted to the pharmacogenetic profile [14]. Beside the PGx variability, also other individual factors such as age, co-morbidities or co-medication via CYP3A4 interaction need to be considered to improve drug treatment and prevent ADRs due to anticoagulation [15,16].

The aim of the IDrug study was to compare effects of an individualized risk assessment for ADRs with a standardized risk assessment to improve safety of drug treatment in patients that are at high risk for bleeding and thromboembolic ADRs, thereby focusing on older, multi-morbid and multi-medicated patients with the intake of antithrombotic drugs.

## 2. Results

In total, *N* = 365 patients were enrolled in the IDrug study and randomized into the individualized or the standardized risk assessment group. Of those, *N* = 340 patients received the respective individualized or standardized risk assessment during visit one, which formed the intention to treat (ITT) cohort. Of those patients, *n* = 273 were followed-up according to the study plan (per protocol (PP) cohort). Within the individualized risk assessment group 80.2% (*n* = 134) and in the standardized risk assessment group 80.3% (*n* = 139) completed the whole follow-up according to the study plan. Supplement 1 lists reasons for dropping out of the ITT cohort (Table S1). Table 1 shows characteristics of the total ITT cohort and stratified to standardized and individualized risk assessment groups.

Patients in the standardized risk assessment group were of a median age of 77 years (IQR 72; 81) and, therefore, were older than those in the individualized risk assessment group (median 75 years (70; 78)) and accordingly scored with a median of 70 (50; 90) less concerning physical function based on the results of the SF-36 questionnaire (compared to 80 (60; 91)). All other parameters were equally distributed over both study groups. A high use of drugs was seen in both study groups with a median intake of 13 drugs (8; 18 in the individualized and 8; 19 in the standardized risk assessment group, respectively). Most patients in both groups received only one antithrombotic drug over the whole study time (median 1 (1; 1)), but in some patients a drug was switched or a treatment modified during follow-up. Therefore, results of antithrombotic drug use sums up to more than 100%. Characteristics of the PP cohort were comparable to the ITT cohort and can be found in Table S2.

Unadjusted results of the composite endpoint (at least one bleeding, thromboembolic event or death) and its single parameters are pictured in Table 2. The table gives the unadjusted ORs and 95% CI calculated using the Mantel–Haenszel common odds ratio estimate. For categorical parameters, in which the calculation of ORs was not possible due to rare occurrence, a *p*-value calculated using Chi-squared test is given.

In the unadjusted analysis, there was no significant difference between study groups concerning the composite endpoint, death, bleeding and thromboembolic events in general and most specific events. However, differences in frequencies can be seen. A stroke/ transient ischemic attack was more common in the individualized risk assessment group (*p* = 0.041), but only in the ITT, not in the PP cohort (Table S3) and with a very small sample size (number of events: *n* = 4).

Table 3 shows the adjusted odds for the composite endpoint and separately for a bleeding event, a thromboembolic event, and death for the ITT cohort. Results for the PP cohort can be found in Table S4.

Patients that received an individualized risk assessment had higher odds for the composite endpoint (any of the following: bleeding, thromboembolic event, or death) than patients that received a standardized risk assessment (OR 1.63 [95% CI 1.02–2.63]) with multiple adjusting (Model 3). Being female was significantly associated with higher odds (2.17 [1.27–3.71]). None of the reduced activity phenotypes were associated with the composite endpoint (CYP2C9 OR 0.92 [0.56–1.51], CYP2C19 OR 1.11 [0.65–1.87], and VKORC1 OR 1.33 [0.67–2.65]). The single outcome parameters of the composite endpoint (bleeding event, thromboembolic event, or death) were not significantly more common in the individualized risk assessment group, but all ORs point towards an association with this group ranging from OR 1.06 [95% CI 0.19–6.09] for death to OR 2.13 [95% CI 0.83–5.44] for thromboembolic events.

There was no significant effect in the PP cohort, but ORs for the composite endpoint, bleeding, and thromboembolic event were all > 1 and CIs were large.

### Secondary Analyses

Table 4 shows the adjusted models for the composite endpoint comparing patients in the individualized versus the standardized treatment group including only those patients taking VKA medication in the ITT cohort. Table S5 shows models for the PP cohort respectively.

The OR for the composite endpoint was even more pronounced in the models when including only patients on VKA treatment with an increase in the individualized treatment group (OR 1.99 [1.05–3.76]). Again, neither reduced CYP2C9, CYP2C19 nor VKORC1 activity were associated with the composite endpoint. Effect sizes of parameters were overall comparable in the PP cohort, even though not significant due to the small sample size. Including only patients with DOAC use revealed an OR of 1.52 [0.63–3.69] pointing to the same direction, but not reaching significance neither in the ITT nor the PP cohort (Table S6).

**Figure 1:**
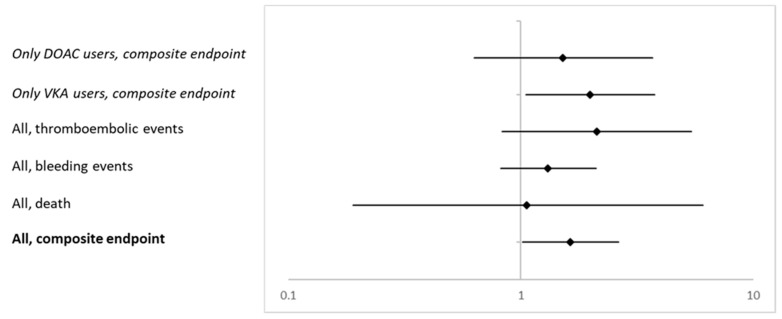
Adjusted ORs and corresponding 95% CI for the composite endpoint and the single items death, bleeding and thromboembolic events in the ITT cohort and for the composite endpoint including only VKA and DOAC users (all Model 3).

Figure 1 summarizes the adjusted ORs and 95% CI for patients in the individualized risk assessment group presenting with a certain outcome in the ITT cohort and in secondary analyses including only VKA and only DOAC users (Model 3).

For around 40–45% of all patients, events were reported via the trigger list, but information was missing in the patient record. Using only the information documented in the patient record, no significant differences between the study groups were detected. A total of 35.3% (*n* = 61) of patients in the standardized risk assessment group and 34.1% (*n* = 57) of the patients in the individualized risk assessment group met the composite endpoint. This resulted in an unadjusted OR of 0.96 [0.61–1.50].

## 3. Discussion

In this analysis of the IDrug study, a pragmatic prospective multicenter randomized controlled trial, we compared effects of an individualized with a standardized risk assessment for preventing ADRs. As a main result of this analysis, patients in the individualized risk assessment group had poorer outcome and higher risk for the composite endpoint meaning experiencing a bleeding and/ or thromboembolic event or death than those in the standardized risk assessment group in adjusted analysis. This effect was even more pronounced in a subgroup analysis of patients on phenprocoumon treatment. However, this effect could not be seen when including only events documented in the patient record or in the unadjusted analysis. The PGx profile seemed to have no impact on the occurrence of ADRs.

An unadjusted increase in the event frequency of 7.3% and an adjusted OR of 1.63 for the composite endpoint in the group with individualized risk assessment are contrary to the study hypothesis that individualized risk information may help to adjust therapy and improve safety in situations with high risk for ADRs. Notably, we saw an association with the composite endpoint, while none of the single items reached statistical significance due to small sample sizes as pictured by large 95% CIs. The unadjusted analysis did not reveal significant differences between the two groups. The participating general practitioners (GP) and their staff reported different experiences with the trigger lists. It might be that women may have a tendency to be more communicative which may have led to a higher reporting rate of (possibly less severe) events [17].

Both the individualized and the standardized risk assessment leaflets contained general information on HAS-BLED and CHA2DS2-VASc scores, drug–drug interactions (DDIs), ageing, renal function, and pharmacogenetic factors, but the individualized risk assessment added extra individualized information per item [18]. In general, patients and GPs were blinded for the study group. However, most GP offices enrolled more than five patients in the study and therefore were confronted with both risk assessments. Therefore, we assume, that blinding became less effective for study group association when enrolling a higher number of patients, which could potentially have been prevented by cluster randomization. Ineffective blinding could have influenced alertness for outcome events in both study groups which may explain why the unadjusted analysis and the analysis using only the patient record data did not detect significant differences between the two groups. In addition, the individualized risk assessment may have led to an increase in doubts about the safety of the current pharmacological treatment both in GPs and patients, which may have resulted in changes in dosing, pharmacologic agent or medication compliance even though the current medication plan was maybe already well balanced.

In clinical practice, alerts integrated into electronic health records are frequently ignored [19,20]. In contrast, our study was a successful collaboration between GPs and clinical pharmacologist and most likely led to a high acceptance rate of recommendations. However, patients were not newly initiated with the drug and therefore, the timing of the risk assessment may have been inappropriate. Retrospectively, using an individualized risk assessment may have been more effective when used upon initiation of an antithrombotic therapy, when serious ADRs might even occur more often [21]. GPs have a high degree of experience with the indication and handling of antithrombotic agents and the benefit of an individualized risk assessment might be highest in patients initiating a new drug treatment such as that performed in the PREPARE trial within the U-PGx project and other PGx implementation projects [22,23].

With more than 50% of all patients meeting the composite endpoint the rate is quite high in our population. Based on other studies one might expect a rate of around 10–15% [21,24]. In general, with a median CHA2DS2-VASc score of four and a median HAS-BLED score of two, one would expect the benefit of the antithrombotic treatment outweighing its risk [25]. The high event rate in our population might be connected to a quite sensitive detection method. Over an observation period of nine months, patients were followed-up three times and a trigger list was used. However, in routine care patients would not have been visited that often, which might explain partly the high observed event rate. Furthermore, using a trigger list we were assessing all kinds of events including, i.e., minor nose bleeding. Bleeding ADRs were most commonly reported in our cohort, but most of the bleeding events were superficial skin or mucosal bleedings. Notably, including only events documented in the patient record, resulted in lower event rates. Meanwhile, the general rate of thromboembolic events seems in line with other studies [26], but was 4.4% higher in the individualized risk assessment group as compared to the standardized risk assessment group.

Associations were even more pronounced in patients taking phenprocoumon. This might be seen in line with a study showing adults aged 75 years and older spending more time out of the therapeutic range (time in therapeutic range, TTR) when genotype-based dosing was used [27]. TTR usually correlates negatively with hemorrhages and thromboembolic rates [26]. This might explain the higher risk for meeting the composite endpoint of bleeding and/ or thromboembolic events or death in our cohort. In another analysis performed with this cohort, we concluded that the genotype had already been empirically respected with INR measurements [14]. Therefore, an added risk assessment might have irritated GPs’ routine care and led to an increase in the composite endpoint. Nevertheless, the risk for adverse events might be even higher in patients without any anticoagulation where an anticoagulation is indicated [28]. Thus, an improved risk assessment might have the potential to initiate a better anticoagulation in older adults.

We did not find an association of a genotype-predicted phenotype with the composite endpoint. This might be seen in contrast to a recent analysis of emergency department admissions that showed a trend towards a combined PGx risk profile of low activity CYP2C9 and VKORC1 genotypes being associated with phenprocoumon-induced bleeding ADRs [29]. However, we included any type of bleeding and thromboembolic events, and a different result might be expected with only including serious ADRs. In addition, compared to the VKA warfarin, CYP3A4 next to CYP2C9 plays a major role in phenprocoumon metabolism. While frequencies of low activity metabolizer phenotypes were in general in line with reported frequencies in European populations [30,31], due to the small sample size absolute numbers of those phenotypes were low. Drug–drug interactions (DDI) might be even more relevant than the PGx profile per se [32]. This cohort was highly multi-medicated with a median intake of 13 drugs per person, although all medications including over the counter and dermatological products were counted. As this population was a cohort of multi-morbid, older adults, the prevalence of potential DDIs with more than 80% of patients was quite high and mostly involving antithrombotic drugs and non-steroidal anti-inflammatory drugs increasing the risk for bleeding [33]. Likewise, pharmacodynamic DDI, e.g., taking several antithrombotic drugs would obviously increase the risk for bleedings [34]. However, the median intake of antithrombotic drugs over the full study period was one and we adjusted the regression analyses for the number of antithrombotic drugs taken.

A strength of this study is the pragmatic design delivering real world data on drug safety in multi-morbid older adults on antithrombotic treatment and good characterization of individual risk factors such as PGx. However, the importance of PGx in age, in particular in the context of DDIs potentially leading to phenoconversion needs to be further studied [35,36]. While most studies enroll patients as older adults basing on the calendrical age (e.g., aged 65 years and older), we used a clinical estimate using multi-morbidity as inclusion criterion that correlated with multi-medication. Therefore, this cohort is formed by a group of clinically relevant older adults [37]. Even though the targeted sample size was not met, this is one of the bigger cohort studies in GP offices implemented in routine care, where the study setting is challenging [38,39] and therefore, delivers precious insight in clinical reality.

Still, the major limitations of this analysis accompany the use of trigger lists and that the calculated sample size of the study was not met [18]. The results need to be interpreted in this light. While the study design was oriented at clinical trial designs, the effort for enrollment and follow-up of the single patients and the single GP offices was high, in particular considering the multi-morbidity of the patients. Another limitation derives from the study design providing risk assessments in patients already on drug treatment. Thereby, the risk assessment especially in the individualized risk assessment group might have led to medication changes that would not have occurred without the risk assessment.

## 4. Materials and Methods

### 4.1. Study Design

Data of the individualized versus standardized risk assessment in patients at high risk for adverse drug reactions (IDrug; trial registration: German Clinical Trials Register: DRKS00006256) was analyzed. The IDrug study is a pragmatic prospective multicenter randomized controlled trial comparing the effect of an individualized versus a standardized risk assessment for reducing ADRs. Study design and information on enrollment is published elsewhere [18]. In brief, patients that were taking an antithrombotic drug together with at least one other regular medication were enrolled in general practitioner (GP) offices and randomized to receive either an individualized or a standardized risk assessment concerning their individual risk for an ADR respecting age, renal function, pharmacogenetics, drug–drug interactions, and bleeding- and thromboembolic risk factors. All patients were followed-up for nine months. The IDrug-study initially attempted to enroll *N* = 960 patients [18] with an assumed event rate of 10% and a potential reduction by 5%.

### 4.2. Study Population

Inclusion criteria of the IDrug study were patients aged 60 years and older, that had more than two concomitant diseases concerning at least two organ systems (multi-morbidity), took at least one antithrombotic drug and at least one additional drug continuously. While usually adults starting by the age of 65 years are considered as older, frailty of patients might differ largely in this age group. We chose a more clinically-relevant cohort with adding multi-morbidity as inclusion criterion. For study inclusion, the intake of a VKA (phenprocoumon or warfarin), of a DOAC (rivaroxaban, apixaban, or dabigatran), and of a P2Y12 inhibitor (clopidogrel or ticagrelor), was counted as antithrombotic drug. Patients were followed-up for nine months summing up to four visits in total.

All patients agreed to participate in the study and provided written informed consent. Patients were enrolled between September 2014 and March 2017 in GP offices in the area of Bonn, Cologne, and Rhine-Sieg-district, in Germany.

The IDrug study was approved by the Ethics Committees of the University of Bonn, of the Medical Association of North Rhine and of the Medical Association of Rhineland-Palatinate.

### 4.3. Study Centers

In total, *n* = 43 GP practices (here termed study centers) enrolled patients into the IDrug study. The number of patients enrolled per study center ranged between 1 and 50. Study centers were grouped according to the number of patients enrolled in the following way: overall, 17.4% of patients (*n* = 59) were enrolled in a study center enrolling between 1 and 5 patients, 20.0% of patients (*n* = 68) were enrolled in a study center enrolling between 6 and 10 patients, 17.6% of patients (*n* = 60) were enrolled in a study center enrolling between 11 and 15 patients, 14.7% of patients (*n* = 50) were enrolled in a study center enrolling between 16 and 20 patients, 6.5% of patients (*n* = 22) were enrolled in a study center enrolling between 21 and 25 patients, and 23.8% of patients (*n* = 81) were enrolled in a study center enrolling 26 patients and more.

### 4.4. Intervention

After enrollment patients were randomized to either receive an individualized or a standardized risk assessment. Age, renal function (creatinine-based glomerular filtration rate (GFR)), genotyping results for CYP2C9, CYP2C19, and VKORC1, potential drug–drug interactions (DDI), and results of the HAS-BLED and the CHA2DS-VASc-scores were used to create an individualized risk assessment by a medical doctor in clinical pharmacology training and supervised by a specialist in clinical pharmacology [18,33], therefore, offering a quality controlled and standardized assessment.

Leaflets were printed and sent to the GP offices. At the initial visit, patients gave their informed consent and all data (lab results, INR value, etc.) were documented. On the next visit, the GP handed out the leaflet with the risk assessment and gave a thorough explanation to the patient. The group with the standardized risk assessment received a look-a-like leaflet, in which single points were mentioned, but in a general and non-personalized way. This leaflet was also explained by the GP [18]. Therefore, the standardized risk assessment group received treatment with standard of care. This might also include DDI assessment or clinical scorings but may depend on clinical routine of the single GPs who tend to use their thorough knowledge of the patients history and habit as a basis of clinical decision making as much as scoring tools.

### 4.5. Data Collection

On study enrollment, age, renal function (GFR), genotyping results for CYP2C9, CYP2C19, and VKORC1, potential drug–drug interactions (DDI), and results of the HAS-BLED and the CHA2DS-VASc-scores were collected for all patients. The patient’s medical history and current drug intake, including over-the-counter medication was documented. Further—sex, weight, height, blood test results, alcohol consumption, smoking status, highest educational degree, and antithrombotic treatment regimen were collected. All patients answered the SF-36 and a questionnaire about drug adherence. In addition, GPs usually had an overview over prescription frequency of single drugs in clinical routine.

### 4.6. Laboratory Methods

An EDTA-blood sample was drawn on enrollment visit from each patient and transferred to the central study laboratory for genotyping. DNA was extracted manually by the High Pure PCR Template Preparation Kit and amplified and detected using real-time-PCR with a LightCycler^®^ 480 instrument (both Roche Diagnostics GmbH, Mannheim, Germany). Genotyping was performed for rs1799853 (CYP2C9*2), rs1057910 (CYP2C9*3) [31], rs4244285 (CYP2C19*2), rs12248560 (CYP2C19*3) [30], rs9934438 (VKORC1 1173C > T) [40] and rs9923231 (VKORC1 1639G > A) [41] using LightMix Kit human reagents (Cat-No 40-0298-32, 40-0304-32, 40-0302-64) from TIB Molbiol GmbH (Berlin, Germany). Melting curve analyses of fluorescent real-time-PCR amplification products were used for allelic identification. If no mutation for CYP2C9 and CYP2C19 was detected, wild type carrier status and extensive metabolism phenotype was assumed. Mutation in one allele was associated with intermediate, mutations in both alleles with slow (poor) metabolism phenotypes. Similarly, double mutations in VKORC1 were considered as slow (poor) metabolism phenotypes. DNA extraction and genotyping was performed by the Institute of Clinical Chemistry and Clinical Pharmacology of the University Bonn.

### 4.7. Phenotype Assessments

For all patients, phenotypes were extrapolated from genotypes. For CYP2C9 and CYP2C19 all patients carrying at least one reduced function allele (*2 or *3) were summarized as having a reduced activity phenotype (*2/*2, *3/*3, *2/*3, *1/*2, *1/*3) in this analysis. The absence of any *2 and the *3 allele was considered a wild-type leading to normal enzyme function. In case of VKORC1, patients carrying at least one C allele (VKORC1 1173C > T) were respected as having reduced clotting activity. The absence of a C allele was interpreted as wild-type carrier status and normal clotting activity.

### 4.8. Antithrombotic Treatment

Data on antithrombotic and drug treatment, and diagnoses were updated on each study visit. A patient was considered to take a specific drug in these analyses, if its use was reported in at least one study visit. To control the analyses for the summative risk of antithrombotic drug intake, a median number of antithrombotic drugs taken over the whole time of the study was calculated per patient. Therefore, the intake of a VKA (phenprocoumon or warfarin), of a DOAC (rivaroxaban, apixaban, or dabigatran), of ASA, of a P2Y12 inhibitor (clopidogrel or ticagrelor), and the administration of a heparin (heparin or low-molecular-weight heparin) was counted.

### 4.9. Study Outcome

On each study visit, patients were asked together with their GP about specific bleeding and thromboembolic events (yes, no) using a trigger list. Within this list, there was an option to report any other bleeding or thromboembolic event not specified in the list. If at least one point of the list was answered with yes, this was considered an event during follow-up. In a next analysis, only reported events documented in the patient record were included in the analysis in order to improve documentation quality. A composite endpoint was used, defined as the occurrence of at least one bleeding, or at least one thromboembolic event, or death during follow-up.

### 4.10. Randomization, Allocation to Study Arm, and Blinding

Randomization was conducted by the Biostatistics Unit of the Federal Institute for Drugs and Medical Devices in Germany. Participating GP offices sent lists of eligible patients. From these lists, patients were randomly selected for participation. Patients were enrolled by GP offices. Then, patients were randomized to receive an individualized or a standardized risk assessment. Randomization was stratified by GP office and sex [18]. According to the allocated study arm, the risk assessment was conducted in the Research Department of the Federal Institute for Drugs and Medical Devices in Germany. Risk assessments were sent out to the GP offices. GPs did not become informed about the study arm allocation of the patients. After finishing data plausibility checks and query management and closing the database, the allocation to study groups was implemented in the study database.

### 4.11. Statistical Analysis

The intention to treat (ITT) cohort was used for all analyses and, where applicable sensitivity analyses were conducted in the per protocol (PP) cohort (Supplement 1).

Descriptive analyses were conducted for the whole study sample and compared between the intervention and the control group. Continuous parameters were checked for normality using Kolmogorov-Smirnov test. Non-normally distributed continuous parameters are presented as median and interquartile ranges (IQR; Q1, Q3), while normally distributed variables are presented as means with standard deviation (SD). Categorical parameters are presented in absolute numbers and percentages. Continuous parameters were compared using a Mann-Whitney test and categorical parameters using a Chi-squared test.

According to the study plan, a composite endpoint was used. Subgroup analyses were performed for the single items of the composite endpoint (at least one bleeding event and specific bleeding events (yes, no), at least one thromboembolic event and specific thromboembolic events (yes, no), and death (yes, no)). The results of the individualized and the standardized risk assessment group for the composite endpoint were compared using Chi-squared test. The Mantel-Haenszel common odds ratio estimate was used to describe unadjusted odds ratios (OR) and 95% confidence intervals (CI). The distribution of number of events between groups was checked for normality using Kolmogorov–Smirnov. As these were normally distributed, groups were compared using a *t*-Test.

Logistic regression analyses were used for adjusting effects of the type of risk assessment (individualized vs. standardized) using different models. Model 1 included age (continuous) and sex (female: yes, no). Model 2 included age, sex, educational degree (categorical), GFR (continuous), number of antithrombotic drugs taken (continuous), HAS BLED score (continuous), CHA2DS2 VASc score (continuous), number of patients enrolled per study center (categorical), and time in study (continuous). Finally, Model 3 included age, sex, educational degree (categorical), GFR (continuous), number of antithrombotic drugs taken (continuous), HAS BLED score (continuous), CHA2DS2 VASc score (continuous), number of patients enrolled per study center (categorical), time in study (continuous), CYP2C9 phenotype (categorical reduced activity), CYP2C19 phenotype (categorical reduced activity), and VKORC1 phenotype (categorical reduced activity). The composite endpoint was used and sensitivity analyses were conducted on bleeding and thromboembolic events and deaths, separately. The time in study was not used as a parameter for analyzing the outcome death in Models 2 and 3 as death reduced the time in study.

Secondary analyses were conducted for the composite endpoint including first, only patients with reported VKA use and second reported DOAC use over study time. For adjustment, the number of antithrombotic drug intake in Models 2 and 3 was calculated without the respective drug class (e.g., number of antithrombotic drugs used excluding VKAs).

Another additional analysis compared the individualized vs. the standardized group using the Mantel–Haenszel common odds ratio estimate including only events from the patient record.

A *p*-value < 0.05 was considered significant. Statistical analyses were conducted with IBM^®^ SPSS^®^ Statistics (Version 25, IBM Inc., Armonk, NY, USA).

## 5. Conclusions

In conclusion, this study underlines the importance of risk assessments for altering safety of drug treatment. While GPs are open to risk assessments and modifying drug treatment in older adults in collaboration with clinical pharmacologists the effectiveness of this intervention and the appropriate time point for its implementation needs further investigations.

## Figures and Tables

**Table 1:** Characteristics of the total population and stratified according to individualized and standardized risk assessment groups (*N* = 340).

Parameter	Missing, *n* (%)	Total Population, *N* = 340	Individualized Risk Assessment Group, *n* = 167	Standardized Risk Assessment Group, *n* = 173	*p*-Value
Age (years), median (IQR)	-	75 (71; 80)	75 (70; 78)	77 (72; 81)	**0.002**
Sex (female), *n* (%)	-	138 (40.6)	65 (38.9)	73 (42.2)	0.539
Number of drugs, median (IQR)	-	13 (8; 18)	13 (8; 18)	13 (8; 19)	0.955
HAS BLED (score), median (IQR)	-	2 (1; 3)	2 (1; 3)	2 (1; 3)	0.653
CHA2DS2 VASc (score), median (IQR)	-	4 (3; 5)	4 (3; 5)	4 (3; 5)	0.432
SF-36 score, median (IQR)					
Vitality	4 (1.2)	65 (50; 75)	65 (50; 80)	63 (45; 75)	0.212
Physical functioning	4 (1.2)	75 (55; 90)	80 (60; 91)	70 (50; 90)	**0.017**
Bodily pain	4 (1.2)	80 (52; 100)	84 (52; 100)	74 (52; 100)	0.672
General health perception	5 (1.5)	65 (50; 67)	65 (52; 77)	62 (49; 77)	0.531
Physical role functioning	5 (1.5)	100 (50; 100)	100 (50; 100)	100 (50; 100)	0.320
Emotional role functioning	5 (1.5)	100 (100; 100)	100 (100; 100)	100 (100; 100)	0.679
Social role functioning	4 (1.2)	100 (88; 100)	100 (97; 100)	100 (88; 100)	0.670
Mental health	4 (1.2)	84 (68; 92)	84 (71; 92)	84 (68; 92)	0.532
Time in study (days), median (IQR)	-	277 (259; 300)	279 (261; 302)	273 (254; 294)	0.062
GFR (mL/min/1.73m^2^)	4 (1.2)	66.2 (51.7; 81.3)	67.4 (52.6; 81.5)	66.2 (51.3; 82.3)	0.424
Renal function, *n* (%)	-				0.240
GFR ≥ 90		32 (9.5)	14 (8.5)	18 (10.5)	
GFR 60–<90		178 (53.0)	91 (55.2)	87 (50.9)	
GFR 30–<60		119 (35.4)	59 (35.8)	60 (35.1)	
GFR 15–<30		5 (1.5)	0 (0)	5 (2.9)	
GFR < 15		2 (0.6)	1 (0.6)	1 (0.6)	
Highest educational degree, *n* (%)	19 (5.6)				0.925
Major school diploma		180 (56.1)	89 (56.3)	91 (55.8)	
Secondary school diploma		60 (18.7)	30 (19.0)	30 (18.4)	
Technical college diploma		16 (5.0)	8 (5.1)	8 (4.9)	
High school diploma		21 (6.5)	9 (5.7)	12 (7.4)	
College degree		43 (13.4)	22 (13.9)	21 (12.9)	
No diploma		1 (0.3)	0 (0)	1 (0.6)	
Number of antithrombotic drugs used, median (IQR)	-	1 (1; 1)	1 (1; 1)	1 (1; 1)	0.883
Antithrombotic drug use, *n* (%)					
VKA	-	209 (61.5)	103 (61.7)	106 (61.3)	0.997
DOAC		101 (29.7)	49 (29.3)	52 (30.1)	0.976
ASA		22 (6.5)	11 (6.6)	11 (6.5)	0.995
P2Y_12_-inhibitor		53 (15.6)	28 (16.8)	25 (14.5)	0.831
PPI use, *n* (%)	-	168 (49.4)	78 (46.7)	90 (52.0)	0.327
Statin use, *n* (%)	-	187 (55.0)	92 (55.1)	95 (54.9)	0.974
CYP2C19 phenotype, *n* (%)	-				0.911
NM		241 (70.9)	120 (71.9)	121 (69.9)	
IM		87 (25.6)	41 (24.6)	46 (26.6)	
PM		12 (3.5)	6 (3.6)	6 (3.5)	
CYP2C9 phenotype, *n* (%)	-				0.488
NM		223 (65.6)	108 (64.7)	115 (66.5)	
IM		110 (32.4)	54 (32.3)	56 (32.4)	
PM		7 (2.1)	5 (3.0)	2 (1.2)	
VKORC1 phenotype, *n* (%)	-				0.724
Normal		295 (86.8)	146 (87.4)	149 (86.1)	
Poor		45 (13.2)	21 (12.6)	24 (13.9)	

IQR: interquartile range, GFR: glomerular filtration rate, VKA: vitamin-K-antagonist, DOAC: directly acting oral anticoagulants, ASA: acetylsalicylic acid, CKD: chronic kidney disease. Significant findings in bold text.

**Table 2:** Frequencies and unadjusted odds of study endpoints (*N* = 340).

Endpoints	Total Population, *N* = 340	Individualized Risk Assessment Group, *n* = 167	Standardized Risk Assessment Group, *n* = 173	OR [95% CI]	*p*-Value
Composite endpoint, *n* (%)	195 (57.4)	102 (61.1)	93 (53.8)	1.35 [0.88–2.08]	
Death, *n* (%)	10 (2.9)	4 (2.4)	6 (3.5)	0.68 [0.19–2.47]	
Patients with bleeding event, *n* (%)	182 (53.5)	91 (54.5)	91 (52.6)	1.08 [0.70–1.65]	
Number of bleeding events, mean (SD)	0.68 (0.74)	0.67 (0.72)	0.68 (0.75)		0.887
Skin or mucosal bleeding, *n* (%)	160 (47.1)	76 (45.5)	84 (48.6)	0.89 [0.58–1.36]	
Hematochezia	15 (4.4)	10 (6.0)	5 (2.9)	2.14 [0.72–6.40]	
Hematuria	28 (8.2)	12 (7.2)	16 (9.2)	0.76 [0.35–1.66]	
Muscle or intra-articular bleeding, *n* (%)	6 (1.8)	2 (1.2)	4 (2.3)	0.51 [0.09–2.83]	
Intra-cranial bleeding, *n* (%)	1 (0.3)	1 (0.6)	0 (0)	-	0.308
Intra-ocular bleeding, *n* (%)	8 (2.4)	4 (2.4)	4 (2.3)	1.04 [0.26–4.22]	
Other bleeding, *n* (%)	12 (3.5)	7 (4.2)	5 (2.9)	1.47 [0.46–4.73]	
Patients with thromboembolic event, *n* (%)	25 (7.4)	16 (9.6)	9 (5.2)	1.93 [0.83–4.50]	
Number of thromboembolic events, mean (SD)	0.08 (0.30)	0.11 (0.37)	0.05 (0.22)		0.088
Superficial venous thrombosis, *n* (%)	3 (0.9)	2 (1.2)	1 (0.6)	2.09 [0.19–23.21]	
Deep venous thrombosis, *n* (%)	2 (0.6)	2 (1.2)	0 (0)	-	0.149
Pulmonary embolism, *n* (%)	1 (0.3)	0 (0)	1 (0.6)	-	0.325
Stroke/ TIA, *n* (%)	4 (1.2)	4 (2.4)	0 (0)	-	**0.041**
Myocardial infarction, *n* (%)	2 (0.6)	1 (0.6)	1 (0.6)	1.04 [0.06–16.70]	
Other thromboembolic event, *n* (%)	13 (3.8)	7 (4.2)	6 (3.5)		

Composite endpoint: any of the following death, bleeding event, or thromboembolic event. SD: standard deviation, TIA: transient ischemic attack. Significant findings in bold text.

**Table 3:** Adjusted odds ratios for the individualized risk assessment group compared with the standardized risk assessment group for study endpoints (*N* = 340).

Endpoints	OR [95% CI] Model 1	OR [95% CI] Model 2	OR [95% CI] Model 3
Composite endpoint	**1.63 [1.03–2.60]**	**1.61 [1.00–2.58]**	**1.63 [1.02–2.63]**
Death	1.16 [0.22–6.08]	1.12 [0.20–6.27] *	1.06 [0.19–6.09] *
Bleeding event	1.33 [0.84–2.10]	1.30 [0.81–2.07]	1.31 [0.82–2.11]
Thromboembolic event	2.08 [0.84–5.11]	2.20 [0.87–5.57]	2.13 [0.83–5.44]

Composite endpoint: any of the following death, bleeding event, or thromboembolic event. Model 1: adjusted for age and sex. Model 2: adjusted for age, sex, educational degree, GFR, number of antithrombotic drugs taken, HAS BLED Score, CHA2DS2 VASc Score, number of patients enrolled per study center, and time in study. Model 3: adjusted for age, sex, educational degree, GFR, number of antithrombotic drugs taken, HAS BLED Score, CHA2DS2 VASc Score, number of patients enrolled per study center, time in study, CYP2C9, CYP2C19, and VKORC1 reduced activity phenotypes. * Time in study was not used as a parameter for the outcome death. Significant findings in bold text.

**Table 4:** Secondary analyses for the composite endpoint comparing the individualized with the standardized risk assessment group including only patients with the intake of vitamin-K-antagonists (*n* = 209).

Parameters Included in Models	OR [95% CI] Model 1	OR [95% CI] Model 2	OR [95% CI] Model 3
Individualized risk assessment	**1.88 [1.02–3.44]**	**1.99 [1.06–3.74]**	**1.99 [1.05–3.76]**
Age (years)	**1.07 [1.02–1.12]**	1.05 [0.99–1.11]	1.05 [0.99–1.11]
Sex (female)	**1.90 [1.03–3.53]**	2.02 [0.99–4.13]	2.04 [0.98–4.26]
Educational degree	-	1.07 [0.87–1.32]	1.07 [0.86–1.32]
GFR (mL/min/1.73m^2^)	-	0.99 [0.97–1.01]	0.99 [0.97–1.01]
Antithrombotic drugs taken (number)	-	1.20 [0.71–2.04]	1.25 [0.73–2.14]
HAS BLED (score)	-	0.97 [0.68–1.38]	0.94 [0.65–1.35]
CHA2DS2 VASc (score)	-	1.15 [0.87–1.52]	1.15 [0.86–1.52]
Amount of patients enrolled in study center	-	0.90 [0.75–1.07]	0.89 [0.75–1.07]
Time in study (days)	-	**1.01 [1.00–1.01]**	**1.01 [1.00–1.01]**
CYP2C9 phenotype (IM/ PM)	-	-	0.77 [0.39–1.52]
CYP2C19 phenotype (IM/ PM)	-	-	0.80 [0.39–1.66]
VKORC1 phenotype (reduced)	-	-	1.32 [0.51–3.41]

Composite endpoint: any of the following death, bleeding event, or thromboembolic event. Model 1: adjusted for age, and sex. Model 2: adjusted for age, sex, educational degree, GFR (glomerular filtration rate), number of antithrombotic drugs taken (excluding vitamin-K-antagonists), HAS BLED score, CHA2DS2 VASc score, number of patients enrolled per study center, and time in study. Model 3: adjusted for age, sex, educational degree, GFR (glomerular filtration rate), number of antithrombotic drugs taken (excluding vitamin-K-antagonists), HAS BLED score, CHA2DS2 VASc score, number of patients enrolled per study center, time in study, CYP2C9, CYP2C19, and VKORC1 phenotypes. Significant findings in bold text.

## Data Availability

Data is contained within the article and Supplementary Material.

## Supplementary Materials

The following are available online at www.mdpi.com/article/10.3390/ph14101056/s1, Table S1: reasons for dropping out of the intention to treat cohort (*N* = 340). Table S2: characteristics of the total per protocol population (*N* = 273) and stratified according to individualized and standardized risk assessment group. Table S3: unadjusted risk and frequencies of the composite primary study endpoint (any bleeding event, any thromboembolic event, or death) (*N* = 273). Table S4: adjusted odds ratios for the individualized risk assessment group compared with the standardized risk assessment group for study endpoints in the per protocol cohort (*N* = 273). Table S5: sensitivity analyses for the composite endpoint comparing individualized risk assessment group with the standardized risk assessment group including only patients with the intake of vitamin-K-antagonists in the per protocol cohort (*n* = 167).
