# Supplementary material for: Individualized versus Standardized Risk Assessment in Patients at High Risk for Adverse Drug Reactions (The IDrug Randomized Controlled Trial)–Never Change a Running System?
Source: Pharmaceuticals (Basel). 2021 Oct 18;14(10):1056. doi: 10.3390/ph14101056 (PMC8538435; doi:10.3390/ph14101056)
Supplement: Supplementary file 1 [file pharmaceuticals-14-01056-s001.zip › pharmaceuticals-1403645-supplementary.pdf]

Table S1. Reasons for dropping out of the intention to treat cohort (N = 340).

Parameter	Total population, N = 340	Individualized risk assessment group, n = 167	Standardized risk assessment group, n = 173
Per protocol (PP) cohort, n (%)	273 (80.3)	134 (80.2)	139 (80.3)
Drop out of ITT, *n* (%)	67 (19.7)	33 (19.8)	34 (19.7)
Patients with bleeding event, *n* (%)	182 (53.5)	91 (54.5)	91 (52.6)
Administrational reasons*, *n* (%)	26 (7.6)	11 (6.6)	15 (8.7)
Not appearing for the study visit, *n* (%)	19 (5.6)	11 (6.6)	8 (4.6)
Consent withdrawn, *n* (%)	11 (3.2)	4 (2.4)	7 (4.1)
Reason unknown, *n* (%)	11 (3.2)	7 (4.2)	4 (2.3)

ITT: intention to treat; *administrational reasons in practice office: e.g., visit conducted at wrong time point, practice office closed during follow-up.

Table S2. Characteristics of the total per protocol population (N = 273) and stratified according to individualized and standardized risk assessment group.

Parameter	Missing, n (%)	Total population, N = 273	Individualized risk assessment group, n = 134	Standardized risk assessment group, n = 139	p-value
Age (years), median (IQR)	-	75 (70; 80)	75 (70; 78)	76 (71; 81)	**0.030**
Sex (male), *n* (%)	-	262 (59.3)	79 (59.0)	83 (59.7)	0.899
Number of drugs, median (IQR)	-	14 (9; 19)	14 (8; 19)	14 (9; 20)	0.629
HAS BLED (score), median (IQR)	-	2 (1; 3)	2 (1; 3)	2 (1; 3)	0.916
CHA2DS2 VASc (score), median (IQR)	-	4 (3; 5)	4 (3; 5)	4 (3; 5)	0.537
SF-36 score, median (IQR)					
Vitality	3 (1.1)	65 (50; 76)	70 (50; 78)	60 (45; 78)	0.124
Physical functioning	3 (1.1)	80 (60; 90)	85 (65; 95)	69 (55; 90)	**0.002**
Bodily pain	3 (1.1)	84 (52; 100)	84 (57; 100)	80 (52; 100)	0.500
General health perception	3 (1.1)	67 (52; 77)	67 (56; 77)	62 (50; 77)	0.257
Physical role functioning	3 (1.1)	100 (50; 100)	100 (50; 100)	100 (50; 100)	0.205
Emotional role functioning	3 (1.1)	100 (100; 100)	100 (100; 100)	100 (100; 100)	0.908
Social role functioning	3 (1.1)	100 (100; 100)	100 (100; 100)	100 (94; 100)	0.283
Mental health	3 (1.1)	84 (72; 92)	88 (76; 92)	84 (72; 92)	0.595
Time in study (days), median (IQR)	-	280 (272; 303)	284 (273; 306)	279 (269; 301)	0.050
GFR (mL/min/1.73m^2)	3 (1.1)	67.2 (52.5; 82.7)	68.2 (52.9; 83.5)	66.0 (49.9; 81.4)	0.212
Renal function, *n* (%)	3 (1.1)				0.281
GFR ≥ 90		26 (9.6)	12 (9.1)	14 (10.1)	
GFR 60- < 90		148 (54.8)	75 (56.8)	73 (52.9)	
GFR 30- < 60		91 (33.7)	45 (34.1)	46 (33.3)	
GFR 15- < 30		4 (1.5)	0 (0)	4 (2.9)	

GFR < 15		1 (0.4)	0 (0)	1 (0.7)	
Highest educational degree, *n* (%)	15 (5.5)				0.949
Major school diploma		142 (55.0)	72 (56.3)	70 (53.8)	
Secondary school diploma		49 (19.0)	24 (18.8)	25 (19.2)	
Technical college diploma		15 (5.8)	7 (5.5)	8 (6.2)	
High school diploma		17 (6.6)	8 (6.3)	9 (6.9)	
College degree		34 (13.2)	17 (13.3)	17 (13.1)	
No diploma		1 (0.4)	0 (0)	1 (0.8)	
Number of antithrombotic drugs used, median (IQR)	-	1 (1; 1)	1 (1; 1)	1 (1; 1)	0.972
Antithrombotic drug use, *n* (%)					
VKA	-	167 (61.2)	80 (59.7)	87 (62.6)	0.624
DOAC		77 (28.2)	37 (27.6)	40 (28.8)	0.831
ASA		16 (5.9)	8 (6.0)	8 (5.8)	0.940
$P2Y_{12}$-inhibitor		46 (16.8)	26 (19.4)	20 (14.4)	0.268
PPI use, n (%)	-	139 (50.9)	63 (47.0)	76 (54.7)	0.206
Statin use, n (%)	-	153 (56.0)	75 (56.0)	78 (56.1)	0.981
CYP2C19 phenotype, *n* (%)	-				0.771
NM		192 (70.3)	96 (71.6)	76 (69.1)	
IM		72 (26.4)	33 (24.6)	39 (28.1)	
PM		9 (3.3)	5 (3.7)	4 (2.9)	
CYP2C9 phenotype, *n* (%)	-				0.517
NM		182 (66.7)	85 (63.4)	97 (69.8)	
IM		85 (31.5)	46 (34.3)	40 (28.8)	
PM		5 (1.8)	3 (2.2)	2 (1.4)	
VKORC1 phenotype, *n* (%)	-				0.941
Normal		240 (87.9)	118 (88.1)	122 (87.8)	
Poor		33 (12.1)	16 (11.9)	17 (12.2)	

IQR: interquartile range, GFR: glomerular filtration rate, VKA: vitamin-K-antagonist, DOAC: directly acting oral anticoagulants, ASA: acetylsalicylic acid, CKD: chronic kidney disease. Significant findings in bold text.

Table S3. Unadjusted risk and frequencies of the composite primary study endpoint (any bleeding event, any thromboembolic event, or death) (*N* = 273).

**Endpoint**	**Total population, *N* = 273**	**Individualized risk assessment group, *n* = 134**	**Standardized risk assessment group, *n* = 139**	**OR [95% CI]**	***p*-value**
Composite endpoint, *n* (%)	161 (59.0)	81 (60.4)	80 (57.6)	1.13 [0.70–1.83]	
Death, *n* (%)	8 (2.9)	2 (1.5)	6 (4.3)	0.34 [0.07–1.69]	
Patients with bleeding event, *n* (%)	151 (55.3)	73 (54.5)	78 (56.1)	0.94 [0.58–1.51]	
Number of bleeding events, mean (SD)	0.73 (0.77)	0.70 (0.77)	0.75 (0.78)		0.618

Skin or mucosal bleeding, *n* (%)	131 (48.0)	60 (44.8)	71 (51.1)	0.78 [0.48–1.25]	
Hematochezia	15 (5.5)	10 (7.5)	5 (3.6)	2.16 [0.72–6.50]	
Hematuria	27 (9.9)	11 (8.2)	16 (11.5)	0.69 [0.31–1.54]	
Muscle or intra-articular bleeding, *n* (%)	5 (1.8)	2 (1.5)	3 (2.2)	0.69 [0.11–4.18]	
Intra-cranial bleeding, *n* (%)	1 (0.4)	1 (0.7)	0 (0)	-	0.308
Intra-ocular bleeding, *n* (%)	8 (2.9)	4 (3.0)	4 (2.9)	1.04 [0.25–4.24]	
Other bleeding, *n* (%)	11 (4.0)	6 (4.5)	5 (3.6)	1.26 [0.37–4.22]	
Patients with thromboembolic event, *n* (%)	21 (7.7)	13 (9.7)	8 (5.8)	1.76 [0.71–4.39]	
Number of thromboembolic events, mean (SD)	0.08 (0.32)	0.11 (0.38)	0.06 (0.23)		0.155
Superficial venous thrombosis, *n* (%)	2 (0.7)	2 (1.5)	0 (0)		0.148
Deep venous thrombosis, *n* (%)	1 (0.4)	1 (0.7)	0 (0)	-	0.308
Pulmonary embolism, *n* (%)	1 (0.4)	0 (0)	1 (0.7)	-	0.325
Stroke/TIA, *n* (%)	3 (1.1)	3 (2.2)	0 (0)	-	0.076
Myocardial infarction, *n* (%)	2 (0.7)	1 (0.7)	1 (0.7)	1.04 [0.06–16.76]	
Other thromboembolic event, *n* (%)	12 (4.4)	6 (4.5)	6 (4.3)		

SD: standard deviation, TIA: transient ischemic attack. Significant findings in bold text.

Table S4. Adjusted odds ratios for the individualized risk assessment group compared with the standardized risk assessment group for study endpoints in the per protocol cohort (*N* = 273).

Endpoint	OR [95% CI] Model 1	OR [95% CI] Model 2	OR [95% CI] Model 3
Composite endpoint	1.31 [0.78–2.19]	1.35 [0.79–2.28]	1.35 [0.79–2.30]
Death	0.46 [0.04–4.66]	0.14 [0.01–3.70] *	0.10 [0.00–2.82]*
Bleeding event	1.11 [0.67–1.86]	1.14 [0.67–1.92]	1.13 [0.67–1.93]
Thromboembolic event	1.84 [0.69–4.87]	1.92 [0.69–5.33]	1.84 [0.66–5.16]

Composite endpoint: any of the following death, bleeding event, or thromboembolic event. Model 1: adjusted for age and sex. Model 2: adjusted for age, sex, educational degree, GFR, number of antithrombotic drugs taken, HAS BLED Score, CHA2DS2 VASc Score, number of patients enrolled per study center, and time in study. Model 3: adjusted for age, sex, educational degree, GFR, number of antithrombotic drugs taken, HAS BLED Score, CHA2DS2 VASc Score, number of patients enrolled per study center, time in study, CYP2C9, CYP2C19, and VKORC1 phenotypes. *Time in study was not used as a parameter for the outcome death. Significant findings in bold text.

Table S5. Sensitivity analyses for the composite endpoint comparing individualized risk assessment group with the standardized risk assessment group including only patients with the intake of vitamin-K-antagonists in the per protocol cohort (*n* = 167).

Parameters included in models	OR [95% CI] Model 1	OR [95% CI] Model 2	OR [95% CI] Model 3
Individualized risk assessment	1.75 [0.89–3.46]	1.89 [0.93–3.84]	1.85 [0.90–3.79]
Age (years)	1.07 [1.01–1.12]	1.05 [0.98–1.12]	1.05 [0.99–1.12]
Sex (female)	1.90 [0.95–3.80]	2.11 [0.93–4.78]	2.21 [0.94–5.17]
Educational degree	-	1.08 [0.85–1.37]	1.07 [0.85–1.36]

GFR (mL/min/1.73m^2)	-	0.99 [0.97–1.01]	0.99 [0.97–1.01]
Antithrombotic drugs taken (number)	-	1.58 [0.82–3.05]	1.64 [0.84–3.21]
HAS BLED (score)	-	1.00 [0.68–1.47]	0.96 [0.65–1.43]
CHA2DS2 VASc (score)	-	1.08 [0.79–1.49]	1.05 [0.76–1.46]
Amount of patients enrolled in study center	-	0.93 [0.76–1.13]	0.92 [0.75–1.12]
Time in study (days)	-	1.00 [0.99–1.01]	1.00 [0.99–1.01]
CYP2C9 phenotype (IM/ PM)	-	-	0.79 [0.36–1.70]
CYP2C19 phenotype (IM/PM)	-	-	0.71 [0.31–1.60]
VKORC1 phenotype (reduced)	-	-	1.96 [0.58–6.68]

Composite endpoint: any of the following death, bleeding event, or thromboembolic event. Model 1: adjusted for age, and sex. Model 2: adjusted for sex, educational degree, GFR (glomerular filtration rate), number of antithrombotic drugs taken (excluding vitamin-K-antagonists), HAS BLED score, CHA2DS2 VASc score, number of patients enrolled per study center, and time in study. Model 3: adjusted for age, sex, educational degree, GFR (glomerular filtration rate), number of antithrombotic drugs taken (excluding vitamin-K-antagonists), HAS BLED score, CHA2DS2 VASc score, number of patients enrolled per study center, time in study, CYP2C9, CYP2C19, and VKORC1 phenotypes. Significant findings in bold text.

Table S6. Secondary analyses for the composite endpoint comparing individualized risk assessment group with the standardized risk assessment group including only patients with the intake of direct oral anticoagulants (n = 101) in the intention to treat (ITT, N = 101) and the per protocol (PP, N = 77) cohort.

**Parameters included in models**	**OR [95% CI] Model 1^{ITT}**	**OR [95% CI] Model 1^{PP}**	**OR [95% CI] Model 2^{ITT}**	**OR [95% CI] Model 2^{PP}**	**OR [95% CI] Model 3^{ITT}**	**OR [95% CI] Model 3^{PP}**
Individualized risk assessment	1.54 [0.67–3.51]	0.96 [0.38–2.44]	1.42 [0.60–3.35]	0.92 [0.34–2.46]	1.52 [0.63–3.69]	0.94 [0.34–2.58]
Age (years)	1.02 [0.96–1.08]	1.04 [0.97–1.11]	1.02 [0.95–1.09]	1.06 [0.98–1.15]	1.01 [0.94–1.09]	1.06 [0.97–1.15]
Sex (female)	1.39 [0.59–3.30]	1.34 [0.49–3.64]	1.83 [0.68–4.95]	1.89 [0.58–6.11]	2.05 [0.74–5.72]	2.00 [0.60–6.65]
Educational degree	-	-	1.06 [0.78–1.44]	1.07 [0.75–1.53]	1.07 [0.79–1.48]	1.01 [0.75–1.62]
GFR (mL/min/1.73m^2)	-	-	0.99 [0.97–1.02]	1.01 [0.98–1.04]	0.99 [0.97–1.02]	1.01 [0.98–1.04]
Antithrombotic drugs taken (number)	-	-	0.96 [0.46–2.01]	1.15 [0.43–3.05]	0.98 [0.46–2.09]	1.19 [0.44–3.20]
HAS BLED (score)	-	-	1.51 [0.84–2.74]	1.30 [0.65–2.60]	1.40 [0.74–2.63]	1.16 [0.56–2.44]
CHA2DS2 VASc (score)	-	-	0.91 [0.66–1.26]	0.86 [0.59–1.23]	0.94 [0.67–1.32]	0.88 [0.60–1.30]
Amount of patients enrolled in study center	-	-	0.86 [0.68–1.10]	0.94 [0.70–1.25]	0.86 [0.67–1.12]	0.92 [0.69–1.24]
Time in study (days)	-	-	1.01 [1.00–1.01]	0.99 [0.98–1.01]	1.00 [1.00–1.01]	0.99 [0.97–1.01]
CYP2C9 phenotype (IM/ PM)	-	-	-	-	0.74 [0.27–2.01]	0.66 [0.21–2.14]
CYP2C19 phenotype (IM/PM)	-	-	-	-	1.60 [0.66–3.85]	0.87 [0.30–2.50]

VKORC1 phenotype (reduced)	-	-	-	-	1.54 [0.33–7.23]	1.66 [0.22–12.46]

Composite endpoint: any of the following death, bleeding event, or thromboembolic event. Model 1: adjusted for age, and sex. Model 2: adjusted for age, sex, educational degree, GFR (glomerular filtration rate), number of antithrombotic drugs taken (excluding directly acting oral anticoagulants), HAS BLED score, CHA2DS2 VASc score, number of patients enrolled per study center, and time in study. Model 3: adjusted for age, sex, educational degree, GFR (glomerular filtration rate), number of antithrombotic drugs taken (excluding vitamin-K-antagonists), HAS BLED score, CHA2DS2 VASc score, number of patients enrolled per study center, time in study, CYP2C9, CYP2C19, and VKORC1 phenotypes.
